# Effects of Sodium Chlorophyllin Copper on APO-1 Expression in Bone Marrow Mesenchymal Stem Cells of Rats with Aplastic Anaemia

**DOI:** 10.1155/2022/6792866

**Published:** 2022-04-06

**Authors:** Xin Jin, Guanhai Dai, Liyang Xuan, Meng Zhang, Huifang Jiang, Yang Sui

**Affiliations:** ^1^Department of Clinical Laboratory, Tongde Hospital of Zhejiang Province, Hangzhou City, Zhejiang Province, China 310012; ^2^Institute of Basic Medicine, Zhejiang Academy of Traditional Chinese Medicine, Hangzhou City, Zhejiang Province, China 310000; ^3^Department of Hematology, Tongde Hospital of Zhejiang Province, Hangzhou City, Zhejiang Province, China 310012; ^4^Department of Clinical Laboratory, The Affiliated Suzhou Hospital of Nanjing Medical University, Suzhou Municipal Hospital, Gusu School, Nanjing Medical University, 242 Guangji Road, Suzhou, 215008 Jiangsu, China

## Abstract

**Background:**

Aplastic anaemia (AA) is a highly prevalent blood disorder in the East and Southeast Asian countries, and a proportion of the patients is poorly treated with immunosuppressive agents. This study is aimed at exploring the effects of sodium copper chlorophyllin (SCC) on rats with AA and at providing the theoretical basis for the treatment of AA using traditional Chinese medicine.

**Methods:**

A rat model of AA was induced by combining 5-fluorouracil with busulfan, and different groups were treated with 25 mg/kg cyclosporin A (CsA) and low-, medium-, and high-dose SCC (25-, 50-, and 100-mg/kg; L-, M-, and H-SCC, respectively). A comparative analysis of peripheral blood counts, T-cell subsets, cytokine levels, bone marrow pathology, and APO-1 expression in mesenchymal stem cells in each group was conducted.

**Results:**

SCC can increase the platelet count and haemoglobin concentration in the peripheral blood of AA rats, whereas bone marrow biopsies revealed that the number of nucleated cells and megakaryocytes of SCC-treated rats increased compared with the model group. This was particularly evident in the H-SCC group. As regards the correction of immune function, unlike CsA, which reduced the absolute CD8+ T-cell count, SCC corrected the imbalanced CD4/CD8 ratio by increasing the absolute CD4+ T-cell count, whereas SCC increased the number of regulatory T-cells and reduced the level of interferon-*γ* in AA rats. When comparing the expression of APO-1 in the MSCs, results of the reverse-transcriptase polymerase chain reaction and Western blot analysis showed that SCC can increase the expression of APO-1 both at the mRNA and protein levels.

**Conclusion:**

We found that SCC can improve haematopoietic function and regress immune disorders in AA rats, which enhanced the expression of APO-1 in bone marrow MSCs. This may be one of the mechanisms of SCC in treating AA.

## 1. Introduction

Aplastic anaemia (AA) is a group of diseases characterised by abnormalities in haematopoietic stem cells, damage to the bone marrow (BM) microenvironment, and ultimately haematopoietic exhaustion and blood cytopenia. AA is a highly prevalent blood disorder in East and Southeast Asian countries, and the incidence in Asian countries is two times higher than that in Western countries [1]. Previous studies have revealed that approximately 70% of AA cases are associated with T-cell immune disorder [2], increased cytotoxic T-cell counts, and hypersecretion of inflammatory factors such as interferon- (IFN-) *γ* and tumour necrosis factor- (TNF-) *α*, resulting in immune damage to haematopoietic progenitor cells [3, 4]. Treatment with immunosuppressive agents such as cyclosporin A (CsA), which inhibits T-cell activity and reduces the production of haematopoietic negative regulators by T-cells [5], can provide symptomatic relief in >50% of patients at the first diagnosis; however, some patients still have less than satisfactory outcomes [6, 7].

With the guidance of basic theories, traditional Chinese medicine (TCM) has achieved some success in the treatment of AA [8, 9]; however, owing to the disadvantages of unknown mechanisms and unified standards, the promotion of TCM treatment has been slow. Therefore, it is important to explore the treatment mechanisms of TCM and improve the operability and operational specifications of TCM [10].

Sodium copper chlorophyllin (SCC) is a porphyrin ring consisting of four pyrrole rings and is extracted from silkworm sand. In our previous study, we found that SCC can recover haematopoiesis and modulate the proliferation and differentiation of mesenchymal stem cells (MSCs) in AA mice [11].

Thus, in this study, we aimed to further investigate the effects of SCC on immunomodulatory function and protein expression in the MSCs of AA rats and to explore the possible pharmacological mechanisms of SCC in the treatment of AA.

## 2. Materials and Methods

### 2.1. Animals and Treatments

To induce an AA model, 3–4-week-old healthy Sprague–Dawley rats, weighing 160–200 g (*n* = 36, 18 male and 18 female), were obtained from Hangzhou Medical College. All procedures and animal experiments were approved by the Ethics Committees of the Tongde Hospital of Zhejiang Province (Identification no. HMU [Ethics] 2019-K051).

In total, 36 rats were divided into 6 groups with 6 rats each: normal, model, low-, medium-, high-dose SCC (25, 50, and 100 mg/kg, L-, M-, and H-SCC) and cyclosporine groups (CsA, 25 mg/kg). All rats, except those in the normal group, received 5-fluorouracil (5-FU) and busulfan (BU) to establish an AA model. Rats were given 5-FU (Huadong Medicine, China) injection (150 mg/kg) intraperitoneally on day 1, followed by gavage administration of BU at a dose of 15 mg/kg (Zhejiang Intmedic Co., China) on day 6, once a week for 3 weeks, and different doses of SCC and CsA were administered by gavage for 15 consecutive days once daily, starting on day 10. In the normal and model groups, rats were treated with the same volume of saline solution.

On the day after the final administration, all rats were anaesthetised intraperitoneally with 3% pentobarbital sodium. After the coma was induced, 5 mL of blood was taken from the abdominal aorta and injected into ethylenediaminetetraacetic acid (EDTA) anticoagulation and procoagulation tubes, respectively. One side of the femur was used for BM counting, whereas the femur of one rat from each group was randomly selected and fixed in 10% formaldehyde solution and kept for pathological examination. The other side of the femur was separated for MSC cell culture.

### 2.2. Peripheral Blood Count and T Lymphocyte Subset

White blood cell (WBC) absolute count, platelet (PLT) count, and haemoglobin (HGB) concentrations were measured using a haematology cell counter (BC-6800, Mindray, China) with EDTA-anticoagulated blood. The T-lymphocyte subset was analysed by flow cytometry. First, cell surface staining was performed by taking 50 *μ*L of blood, added with 5 *μ*L each of CD3-PE-Cy5.5 (Clone 1F4, BioLegend), CD4-PE-Cy7 (Clone W3/25, BioLegend), CD8-APC (Clone G28, BioLegend), and CD25-FITC (Clone OX-39, BioLegend) antibodies and incubated for 20 min in the dark. Then, haemolysin, fixative agent, and membrane breaker were added in this order; finally, 5 *μ*L of FOXP3-PE (Clone 150D, BioLegend) was added for 20 min and centrifuged. The supernatant was discarded, and the sample was resuspended. In the flow cytometry assay (NAVIOS, Beckman Coulter, USA), at least 20,000 lymphocytes or 10,000 CD4+ T-cells were collected per sample, and an isotype control was set up to determine the positive expression of the results.

### 2.3. BM Count and Pathology

BM cells in one femur were flushed out with 1 mL of phosphate-buffered saline for cell counting. Meanwhile, one rat from each group was randomly selected for BM pathology examination, and the tissue and metaphysis were removed from the femur and placed in a liquid containing 6% hydrochloric acid and 10% formaldehyde for decalcification and simultaneous fixation. After fixation, the tissue was dehydrated, embedded in paraffin, sectioned, and routinely stained with haematoxylin and eosin, and pathological changes were observed under the microscope by an experienced pathologist.

### 2.4. Detection of Plasma Cytokine

The concentrations of IFN-*γ*, interleukin-6 (IL-6), and TNF-*α* were measured using precoated enzyme-linked immunosorbent assay kits (DAKEWE, China), according to the manufacturer's instructions.

### 2.5. Isolation and Identification of BM MSCs

The BM cavity was repeatedly rinsed 3–5 times with 5 mL of *α*-Minimum Essential Medium (MEM), blown, and dispersed. The supernatant was discarded by centrifugation at 1000 rpm for 5 min; the cells were resuspended with 5 mL of 10% *α*-MEM medium, mixed well, transferred to a culture dish, and incubated at 37°C in a 5% CO_2_-saturated humidity incubator. The medium was changed for the first time after 48 h, and the suspension cells were collected and then changed every 3 days. The monolayer of adherent cells was digested with 0.25% trypsin and 0.02% EDTA in a 1 : 3 ratio for passaging when it was half confluent, labelled as passage 1 (P1), returned to the incubator, and continuously cultured. Cell growth was observed every other day. Cells cultured beyond passage 3 (P3) were digested with trypsin, and the purity of the isolated MSC was determined by CD90-PE (Clone OX-7, BioLegend), CD44-FITC (Clone OX-49, Cedarlane), CD11b/c- PE-Cy5.5 (Clone OX-42, BioLegend), and CD45-PE-Cy7 (Clone OX-1, BioLegend) with flow cytometry.

### 2.6. Reverse-Transcription Polymerase Chain Reaction (RT-PCR)

The total RNA of each sample was isolated using a TRIzol Reagent (Invitrogen, MA, USA) according to the manufacturer's protocol. Reverse transcription was performed using a Prime Script™ RT reagent Kit (TaKaRa, Japan), and 1 *μ*g of RNA was reverse transcribed into cDNA in a 25 *μ*L reaction system and stored at −80°C until use. All oligonucleotide primers were designed using the Perlprimer software and synthesised commercially (Sangon Biotechnology, Shanghai, China). The sequences of the primers are shown in Table 1, and quantitative PCR was performed on the StepOnePlus Real-Time PCR System (Applied Biosystems, MA, USA) using a SYBR Premix EX Taq™ II (TaKaRa, Japan) following the manufacturer's protocol. The two-step PCR reaction conditions were as follows: initial denaturation at 95°C for 30 s, followed by 40 cycles of denaturation at 95°C for 5 s and annealing and extension at 60°C for 30 s. Each sample was tested in triplicate. The relative fold change in gene expression was calculated using the 2^−ΔΔCt^ method.

### 2.7. Western Blot (WB) Analysis

The expression of Apo-1 protein of MSCs was detected by WB analysis. Cells were lysed in RIPA buffer (Beyotime Biotechnology, China). Total protein quantification was performed using the BCA Protein Assay Kit according to the manufacturer's instructions. Then, a capillary-based Simple Western Analysis (ProteinSimple, CT, USA) was used to detect protein abundance according to the manufacturer's protocol. Briefly, 0.6 *μ*g of whole protein sample was loaded into each well, and the size of the separation matrix ranged from 12 to 230 kDa. The target protein was identified using primary antibody-APO1, and glyceraldehyde 3-phosphate dehydrogenase (ProteinTech, IL, USA) was used as a reference control. According to primary antibodies, rabbit secondary antibody provided by the manufacturer was used. Chemiluminescent signals were detected and quantified using Compass Software (ProteinSimple), and the area value was used as the protein expression level.

### 2.8. Statistical Analysis

Statistical analysis was performed using IBM SPSS Statistics for Windows, version 22.0 (IBM Corp., Armonk, USA). Data are expressed as the mean ± standard deviation and *n* (%). Student's *t*-test or one-way analysis of variance was conducted to compare two groups and compare three or more means. *P* values < 0.05 were considered significant.

## 3. Results

### 3.1. SCC Increases HGB and PLT Count in AA Rats

**Figure 1:**
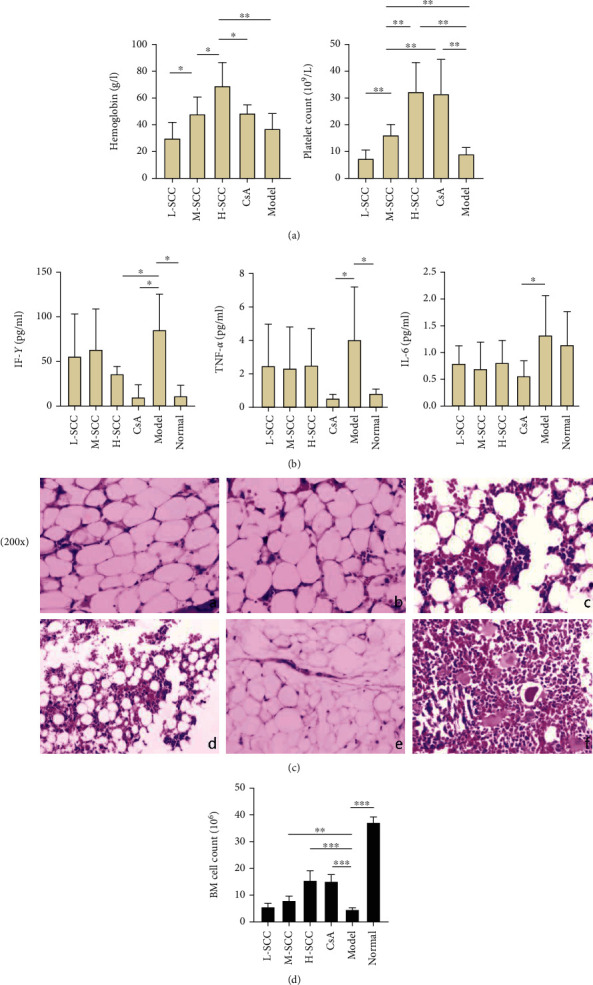
SCC improves hematopoiesis and reduces inflammatory factors in AA rats. (a, b) Comparison of peripheral blood cells and cytokine concentrations in various groups of rats (*n* = 6). Results are presented in the bar charts, data are presented as mean ± SD. Statistically significant differences are indicated by ^∗^*P* < 0.05; ^∗∗^*P* < 0.01; ^∗∗∗^*P* < 0.001. (c) Typical pictures of femoral bone marrow biopsies of rats in each group are shown by H&E staining, 200x. In which, a, b, and c represents model groups treated with SCC at low, medium and high dose, respectively, d for treatment group with CsA, e is the model group, and f is the normal rats.

The HGB level and WBC and PLT counts of rats in the model group and each treated group were lower than those in the normal group (data not shown). When comparing counts in the model and treated groups, the HGB level was higher in the H-SCC group than in model and CsA groups (*P* = 0.005 and *P* = 0.028), and differences were found between the H-, M-, and L-SCC groups (*P* = 0.045, *P* = 0.037). The PLT counts were higher in the M-, H-SCC, and CsA groups than in the model group (*P* = 0.006, *P* = 0.003, *P* = 0.005). Meanwhile, the PLT count in the CsA group was higher than those in the M- and L-SCC groups (*P* = 0.003 and *P* = 0.044), and PLT counts increased with increasing doses in the three SCC treatment groups (*P* = 0.003, *P* = 0.007). However, no difference in WBC was noted between the treated and model groups (Figure 1(a)).

### 3.2. Inhibition of IFN-*γ* Production in AA Rats by SCC

As shown in Figure 1(b), the levels of IFN-*γ* and TNF-*α* in the model group were significantly higher than those in the normal group (*P* = 0.011, *P* = 0.035). The level of IFN-*γ* decreased significantly in the H-SCC and CsA groups compared with the level in the model group (*P* = 0.039, *P* = 0.010), whereas the TNF-*α* level, just in the CsA group, decreased significantly compared with the level in the model group (*P* = 0.024). Moreover, only the IL-6 level was different between the CsA and model groups (*P* = 0.044).

### 3.3. SCC Can Reduce Myelosuppression in AA Rats

In normal rats, the BM was actively proliferating, with numerous nucleated cells, megakaryocytes were easily seen, and haematopoietic stem cells were evenly distributed in the BM cavity. Conversely, in the model group, the proliferation of haematopoietic stem cells was significantly inhibited, and the BM cavity was filled with adipocytes; only a few lymphocytes remained scattered. As shown in Figure 1(c), reduced myelosuppression was found in AA rats of all treated groups, with more nucleated cells and fewer adipocytes in the model group. This phenomenon was particularly evident in the H-SCC- and CsA-treated groups. Regarding BM counts, we found a significant increase in the number of nucleated cells in the M- and H-SCC groups compared with those in the model group (*P* = 0.004, *P* < 0.001).

### 3.4. SCC Increases Th Cells and Regulatory T-Cells (Tregs) in AA Rats

**Figure 2:**
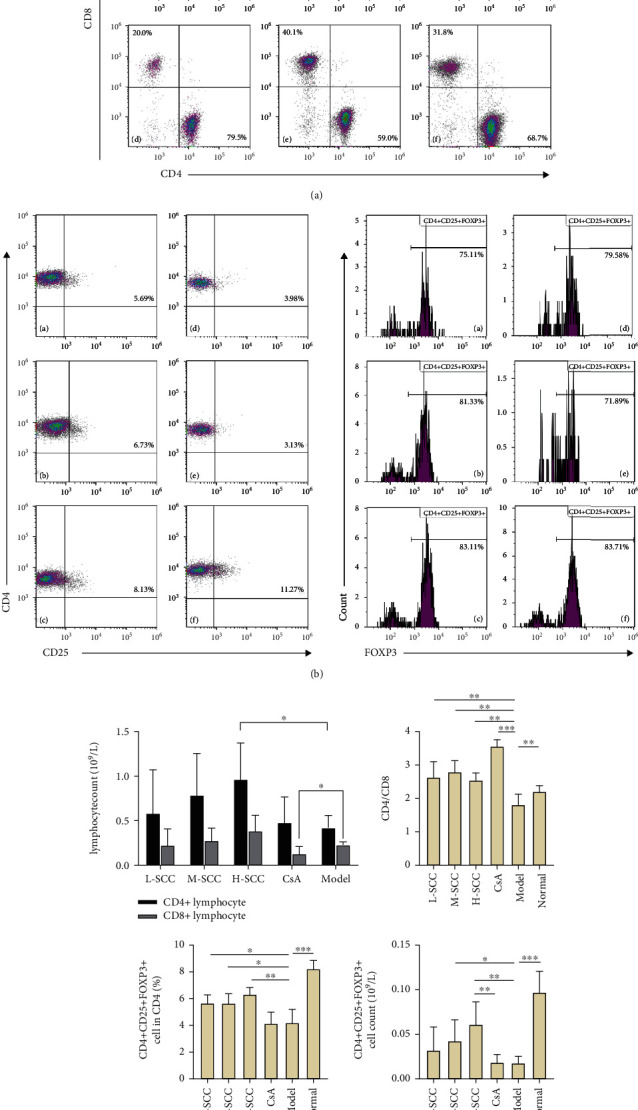
Comparative analysis of peripheral T cell subsets. (a) A representative dot plot of CD8 and CD4 in each group of rats. (b) By flow cytometry, we defined the subpopulation of CD4+CD25+ cells expressing FOXP3 as Treg cells, with the presentation of typical data for different groups. (c) Comparative of CD4/CD8, absolute values of CD4+ and CD8+ cells and the percentages of Treg in rats of each group (*n* = 6); a, L-SCC group; b, M-SCC group; c, H-SCC group; d, CsA group; e, model group; f, normal group. Statistically significant differences are indicated by ^∗^*P* < 0.05; ^∗∗^*P* < 0.01; ^∗∗∗^*P* < 0.001.

Compared with the normal group, the proportion of CD8+ T-cells was significantly higher, and those of CD4+ T-cells, CD4+CD25+FOXP3+ Tregs and CD4/CD8 ratio were significantly lower in the model group. Compared with the model group, the L-SCC, M-SCC, H-SCC, and CsA groups had a higher proportion of CD4+ cells, a lower proportion of CD8+ cells, and a significantly higher CD4/CD8 ratio (*P* = 0.008, *P* = 0.001, *P* = 0.002, *P* < 0.001, respectively). In our analysis of the absolute values of T-cell subsets, the CD4+ cell count was significantly higher in the H-SCC group (*P* = 0.013), and the absolute CD8 cell count was significantly lower in the CsA group than in the model group (*P* = 0.045). The proportion of CD4+CD25+FOXP3+ Tregs was significantly higher in each SCC dose group than in the model group (*P* = 0.017, *P* = 0.023, *P* = 0.002); however, no difference in Tregs was found between the CsA group and the model group. Moreover, the absolute values of Tregs in the H-SCC group were significantly higher than those in the CsA and model groups (Figure 2).

### 3.5. MSC Morphology and Immunophenotype

**Figure 3:**
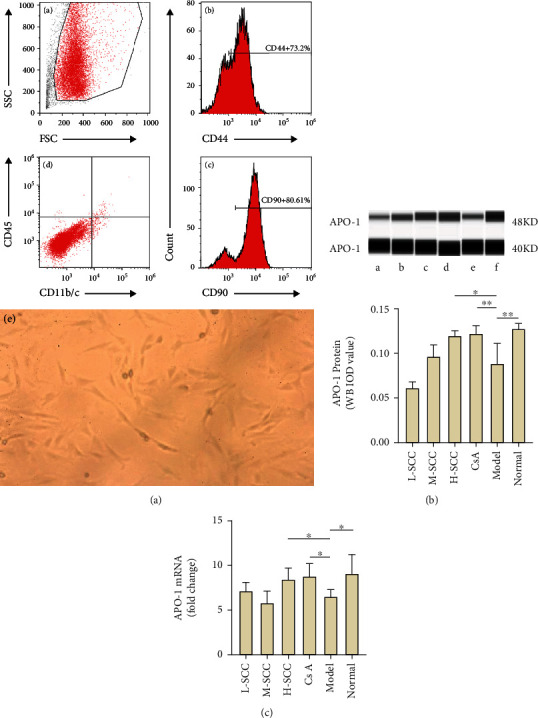
(a) Morphological and immunological identification of MSC; a, remove debris with FSC as well as SSC and circle out the target cells; b and c, MSC positively for CD44 and CD90; d, negative expression of CD45 and CD11b/c; e, typical MSC with spinning cone morphology. (b) Western blot was used to visualize the accumulation of APO-1 proteins in MSC in different groups; a, L-SCC group; b, M-SCC group; c, H-SCC group; d, CsA group; e, model group; f, normal group. Chemiluminescent signals were detected and quantified with Compass software. (c) Comparison of APO-1 protein and mRNA expression on MSC in each group of rats (*n* = 6). Statistically significant differences are indicated by ^∗^*P* < 0.05; ^∗∗^*P* < 0.01.

MSCs in the treatment, model, and normal groups demonstrated a similar spindle-shaped morphology and were immunophenotypically positive for CD44 and CD90 and were negative for CD45 and CD11b/c after cell staining and detecting in flow cytometry, as shown in Figure 3(a). No significant difference was found in the expression of any single marker among the study groups (data not shown).

### 3.6. SCC Enhances APO-1 Expression in MSC

As presented in Figures 3(b) and 3(c), the results of the WB indicated that the expression of APO-1 protein in MSCs was significantly decreased in the model group compared with the expression in the normal group (*P* = 0.003), while the protein expression of APO-1 was increased in the H-SCC and CsA treatment groups compared with the expression in the model group (*P* = 0.011, *P* = 0.009). Meanwhile, similar results were obtained by RT-PCR; the mRNA of APO-1 was higher in the H-SCC and CsA treatment groups than in the model group (*P* = 0.016, *P* = 0.010). These results indicated that SCC could enhance the expression of APO-1 in MSC.

## 4. Discussion

In this study, the peripheral blood HGB concentration, WBC and PLT counts, BM proliferation, and megakaryocyte count were significantly lower in the model group than in the normal group, suggesting that we have completed successful modelling of AA. Meanwhile, peripheral blood counts and BM did not return to normal levels in all treated groups. However, compared with the model group, the M-SCC, H-SCC, and CsA groups can increase the platelet count in the AA rats. As regards improving anaemia, only the H-SCC group showed a difference compared with the model group. Moreover, the HGB level of the H-SCC group was higher than that of the CsA group. The results suggest that SCC can improve the haematopoietic function of AA rats. Meanwhile, among the three doses of SCC, the high dose of 100 mg/kg is the most effective, but further experiments are needed to determine whether the continued increase in SCC concentration can enhance the therapeutic effect. Interestingly, our data show that SCC is better than CsA, a commonly used clinical immunosuppressant, in increasing the HGB concentration, which may open a new way for the clinical treatment of AA.

In recent years, numerous studies have demonstrated that cellular immunity plays a large role in the development of BM failure in AA [12–14], such as overactivation of CD8+ killer T-cells [15] and diminished immunosuppressive function of Tregs [16, 17]. In our study, immune dysfunction in the model rats, including the downregulation of the CD4/CD8 ratio, decreased in CD4+CD25+FOXP3+ Tregs and the secretion of inflammatory factors TNF-*α* and IFN-*γ* increased, which reflected a negative regulation of haematopoiesis [18, 19]. However, although all treatment groups had an increased CD4/CD8 ratio compared with the model group, differences were still found between the SCC and CsA groups.

As an immunosuppressant, CsA suppresses T-cell immune function [20], and we found that the absolute count of CD8 cells and levels of TNF-*α* and IFN-*γ* were significantly reduced in the CsA group compared with those in the model group. Unlike the CsA group, the absolute count of CD8 cells did not change in the H-SCC group; however, the absolute count of CD4 cells and Tregs increased significantly. In the regulation of inflammatory factors, H-SCC also reduced the concentration of IFN-*γ*; however, the effect was weaker than that of the CsA group, and no significant difference was found in the TNF-*α* levels between the SCC and model groups. These results imply that the mechanism of action of SCC in improving haematopoiesis of rats may be different from that of CsA, as CsA achieves the purpose of treatment by suppressing overactivated T-cells, whereas SCC treatment may improve immune regulation.

The BM stroma consists of different populations of haematopoietic and nonhaematopoietic stem cells [21]. Nonhaematopoietic progenitor cells are called MSCs. MSCs are self-renewing and can be induced to differentiate into cells of mesodermal lineages, such as adipocytes, chondrocytes, and osteoblasts [22]. MSCs also play important roles in the occurrence of AA by affecting haematopoietic stem cells and immune cells [23–25]. Apo-1, also known as Fas, is a member of the TNF receptor family and has been broadly studied for its function during development and ability to regulate both ligand-mediated and activation-induced cell death in various cells. The effect of the Fas/FasL system in AA has also been studied [26, 27]. However, these studies have focused on haematopoietic stem cells and rarely on the expression of Fas in MSCs.

Recently, the immunosuppressive effect of MSCs is the hotspot for MSC treatment. Several studies have confirmed that the Fas/FasL system is important for T­cell apoptosis in inflammatory and immune diseases [28–30], and enhancement of Fas/FasL expression is a promising strategy to improve MSC cytotherapy at the molecular level [31]. In our study, we found that SCC could increase the expression of APO-1 at both the transcriptional level of mRNA and protein level in BM MSCs. We think that improving the immunosuppression through the Fas/FasL system is one of the pathways for SCC to treat AA and improve myelopoiesis. However, the study showed that the enhanced Fas/FasL system induces massive apoptosis in Treg B-cells, with high FasL expression, leading to immune hyperactivity and the development of AA [32]. Treg B-cells with high FasL expression are only a small subset of the negative immunomodulatory cells; combined with our results, we consider that SCC may achieve the goal of treating AA by promoting the apoptosis of CD8+ T-cells or inducing naive T-cells to differentiate towards Tregs rather than towards activated T-cells. In this study, we only found this phenomenon in animal models; thus, further experiments are needed. In our next study, we will focus on the immune changes affected by SCC through the Fas/FasL pathway to investigate the pharmacological mechanisms of SCC.

In conclusion, the results of this study revealed that SCC can improve haematopoiesis and regress immune disorders to some degree in AA rats. Moreover, enhancing the expression of APO-1 in bone marrow MSCs may be one of the mechanisms of SCC in treating AA, but this needs further confirmation.

## Figures and Tables

**Table 1:** RT-­PCR primers.

Genes	Primer sequences
GAPDH	Forward: 5′-GACATGCCGCCTGGAGAAAC-3′ Reverse: 5′-AGCCCAGGATGCCCTTTAGT-3′
APO-1	Forward: 5′-GTCCTGCCTCTGGTGCTTGC-3′ Reverse: 5′-CACGAACGCTCCTCTTCAACTCC-3′

## Data Availability

All data relevant to the study are included in the article.

## Abbreviations

AA: Aplastic anaemia
BM: Bone marrow
CsA: Cyclosporin A
IFN-*γ*: Interferon *γ*
IL-6: Interleukin-6
MSC: Mesenchymal stem cell
SCC: Sodium copper chlorophyllin
TCM: Traditional Chinese medicine
TNF-*α*: Tumour necrosis factor-*α*
Tregs: Regulatory T-cells
WBC: White blood cell.
